# Involvement of proapoptotic genes in autophagic cell death induced by irradiation

**DOI:** 10.1038/cddiscovery.2017.68

**Published:** 2017-12-04

**Authors:** Jianrui Song, Xue Zhao, Yi Feng, Sheng Xu, Yanling Zhang, Lixin Wei

**Affiliations:** 1 Tumor Immunology and Gene Therapy Center, Eastern Hepatobiliary Surgery Hospital, The Second Military Medical University, Shanghai 200438, China; 2 Department of Cell and Developmental Biology, University of Michigan, Ann Arbor, MI 48109, USA; 3 Renji Hospital, School of Medicine, Shanghai Jiaotong University, Shanghai 200217, China; 4 Department of Critical Care Medicine, Shanghai General Hospital, Shanghai Jiaotong University, Shanghai 200080, China; 5 Department of Radiotherapy, Eastern Hepatobiliary Surgery Hospital, The Second Military Medical University, Shanghai 200438, China; 6 Department of Biochemistry and Molecular Biology, Soochow University Medical College, Suzhou, Jiangsu 215123, China

## Abstract

Irradiation is widely used in anticancer therapy; however, the efficiency is limited. Most cancer cells have mutations in apoptosis that they can easily escape the apoptosis induced by irradiation. Autophagy has been known as type II programmed cell death that can be activated by irradiation, especially when apoptosis is blocked, but the underlying molecular mechanism is largely unknown. We demonstrated that proapoptotic genes PUMA and Bid are involved in the regulation of autophagic cell death. When wild-type (WT), Bax^−/−^ and PUMA^−/−^ HCT116 cells were exposed to irradiation, we found that, compared with WT, Bax^−/−^ cells showed significantly decreased cell death because of Bax deficiency; however, surprisingly PUMA^−/−^ cells showed significant increase in cell death although the proapoptotic gene PUMA was knocked out. By analyzing apoptosis via Annexin V-fluorescein isothiocyanate (FITC) assay with flow cytometry, both Bax^−/−^ and PUMA^−/−^ cells showed less apoptosis than WT, suggesting the existence of another type of cell death in PUMA^−/−^ cells. Autophagy was then examined in three cell lines by counting the percentage of cells with punctate GFP-LC3. Although all three cell lines showed significantly increased autophagy activity after irradiation, that of PUMA^−/−^ cells was much higher than the other two cell lines, which suggests that PUMA^−/−^ cells may die through autophagy. This was then confirmed by the decreased cell death in PUMA^−/−^ cells when autophagy was blocked by 3-MA. In addition, we also tested the responses of WT and Bid^−/−^ MEFs to irradiation. Bid^−/−^ MEFs but not WT died through autophagy after irradiation. These results imply the involvement of apoptosis-associated genes such as PUMA and Bid in autophagic cell death, which contributes to identifying the molecular mechanism by which autophagy drives cells to death.

## Introduction

Radiotherapy (RT) has been importantly involved in anticancer treatments. Around 50% of cancer patients receive RT at some stage of their treatment, alone or in combination with other treatments such as surgery and/or chemotherapy.^1–4^ Ionizing radiation (IR) is the most commonly used RT, which mainly causes damage by DNA double-strand breaks leading to cell death.^5^ IR successfully helped local control and increased overall survival.^2,6,7^ However, IR is limited and shows poor effect in a significant proportion of high-risk patients who may develop metastasis in several years,^8,9^ which cannot be resolved by simply further dose escalation because of toxicity to adjacent normal tissues. In addition, the resistance of cancer cells to IR causes treatment failure too. Therefore, exploring novel targeted agents to augment the efficiency of RT is in need.

The goal of RT is to completely eliminate cancer cells through initiating cell death programs. IR leads to cell death via apoptosis, which is characterized by DNA fragmentation, vacuolization and nuclear condensation.^10^ Bcl-2 family proteins are known as critical regulators of apoptosis.^11^ These proteins contain one or more of the four conserved motifs, named Bcl-2 homology (BH) domains (BH1, BH2, BH3 and BH4), which are known for their crucial functions.^12^ These Bcl-2 family proteins roughly fall into three subtypes: antiapoptotic subtype that conserve all four BH domains, such as Bcl-2 and Bcl-xL;^13^ proapoptotic subtype with several BH domains called multi-domain apoptosis effectors, including Bax and Bak; and the ones that contain a single BH3 domain called BH3-only apoptosis activators, such as Bid, Bim, Bad and PUMA.^14,15^ They work together to determine the initiation of apoptosis.^12,16^ Researchers have been working on increasing apoptosis to improve RT; however, loss of apoptosis is a frequent event in malignant tumors, which leads to radioresistance. Homozygous deletions or inactivating mutations of Bax have been identified particularly in cancers that arise with defective DNA mismatch repair.^17,18^ However, apoptosis is not the only damage response to IR. Studies show that radiation-induced apoptosis accounts for <20% of cell death.^19,20^

Another type of programmed cell death, autophagy, has been identified as an alternative response to irradiation.^20–23^ Autophagy is a genetically programmed, evolutionarily conserved degradative process that is characterized by sequestration of long-lived cellular proteins and organelles in autophagic vesicles (also named autophagosomes) that are later fused with lysosome to generate autolysosome and are degraded by the cells’ own lysosomal system.^23,24^ The role of autophagy in cancer therapy is controversial; depending on the cell line and the context, autophagy either represents a protective mechanism or contributes to cell death. Autophagy allows cancer cells to degrade proteins and organelles to generate macromolecular precursors, such as amino acids, fatty acids and nucleotides, in order to provide metabolic substrates to enhance survivability and inhibit apoptosis.^25–27^ In this context, blocking autophagy suppresses tumor growth.^25^ Studies have shown that cancer cells use autophagy as an adaptive system to overcome radiotherapeutic stress: autophagy increases in tumor cells in response to radiation and DNA damage, and radioresistance may be associated with autophagy induction.^28–31^ Several studies indicate that pharmacological or genetic inhibition of autophagy can sensitize cancer cells to RT.^32^ Nevertheless, persistent accumulation of autophagic vesicles after high levels of damage may offset the protective effects but lead to eventual autophagocytosis and cell death.^20–22,33,34^ It is shown that heterozygous disruption of Beclin 1 promotes tumorigenesis, but overexpression of Beclin 1 inhibits tumor growth in mice.^35,36^ The former is supported by observations in patients that Beclin 1 is frequently monoallelically lost in human breast, ovarian and other tumors.^37^ Therefore, autophagy is a double-edged sword;^38^ however, the critical molecules that control the divergence of these two opposite functions are far from clear.

In order to elucidate the mechanism by which autophagy leads to cell death, we investigated the response of three isogenic human colon cancer cell lines: WT, Bax^−/−^, and PUMA^−/−^ HCT116, to a serial dose of irradiation and did the comparison. We then determined the biological processes that contribute to the difference between those cell lines. The study was then expanded to normal cell lines of WT and Bid^−/−^ mouse embryonic fibroblasts (MEFs). This study aimed to develop strategies to overcome radioresistance of apoptosis-deficient cancer cells and improve RT in cancer treatment.

## Results

### PUMA deficiency sensitizes HCT116 cells to irradiation

**Figure 1:**
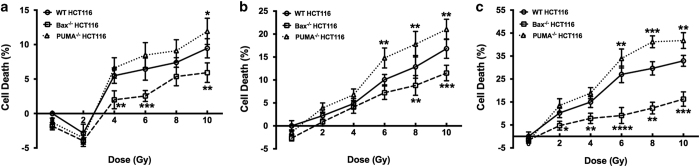
Less cell death in Bax^−/−^ HCT116 but more in PUMA^−/−^ HCT116 when compared with WT cells after irradiation. Human colon cancer cells WT, Bax^−/−^ and PUMA^−/−^ HCT116 were subjected to irradiation for the indicated doses. After incubation for 24 (**a**), 48 (**b**) or 72 h (**c**), cell death assay was performed using CCK-8. Cell death was shown as means±S.D. Statistical significant differences are marked as *P<0.05, **P<0.01, ***P<0.001 or ****P<0.0001.

Irradiation is widely used in colon cancer treatment, but patients respond differently ranging from radiosensitive to radioresistant. In order to identify the underlying contributor for the difference, WT, Bax^−/−^ and PUMA^−/−^ HCT116 cells were subjected to irradiation. Cell death was determined at different time points. All cells showed a time- and dose-dependent toxicity to irradiation, and compared with WT HCT116, Bax^−/−^ cells showed significant decrease in cell death, which likely attributes to the deficiency of Bax, a critical proapoptotic gene (Figure 1). However, surprisingly PUMA^−/−^ cells showed significantly increased cell death although PUMA that is also a proapoptotic gene is deficient (Figure 1). In order to explain this phenotype, we first detected the apoptosis in each cell line and focused subsequent experiments on the dose of 8 Gy at 72 h after irradiation as PUMA^−/−^ cells showed the most significance at this dose and time point.

### Apoptosis is decreased in PUMA^−/−^ HCT116 cells

**Figure 2:**
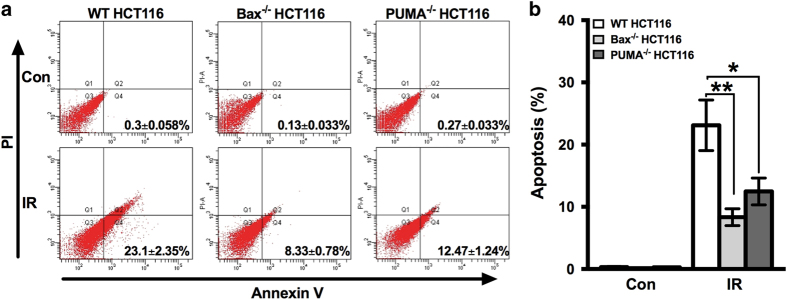
Apoptosis of Bax^−/−^ and PUMA^−/−^ HCT116 cell after irradiation. Irradiation of 8 Gy was given to WT, Bax^−/−^ and PUMA^−/−^ HCT116 cells, and after 72 h of incubation, apoptosis was determined by Annexin-V and PI staining with flow cytometry. Representative flow cytometric plots (**a**) and the quantification (**b**, mean±S.D.) are shown. Con, control, no irradiation. IR, irradiation. Statistical significant differences are marked as *P<0.05 or **P<0.01.

In order to make sure the increased cell death in PUMA^−/−^ HCT116 cells upon irradiation was not resulted from the unexpected increase in apoptosis, because theoretically it should decrease, we determined the apoptosis of WT, Bax^−/−^ and PUMA^−/−^ HCT116 cells, respectively, by Annexin V-FITC assay with flow cytometry. As expected, apoptosis was decreased in both Bax^−/−^ and PUMA^−/−^ cells as a result of losing a proapoptotic gene (Figure 2), indicating that the increased cell death in PUMA^−/−^ HCT116 cells is not attributed to change in apoptosis.

### PUMA knockout sensitizes HCT116 cells to irradiation by inducing autophagic cell death

**Figure 3:**
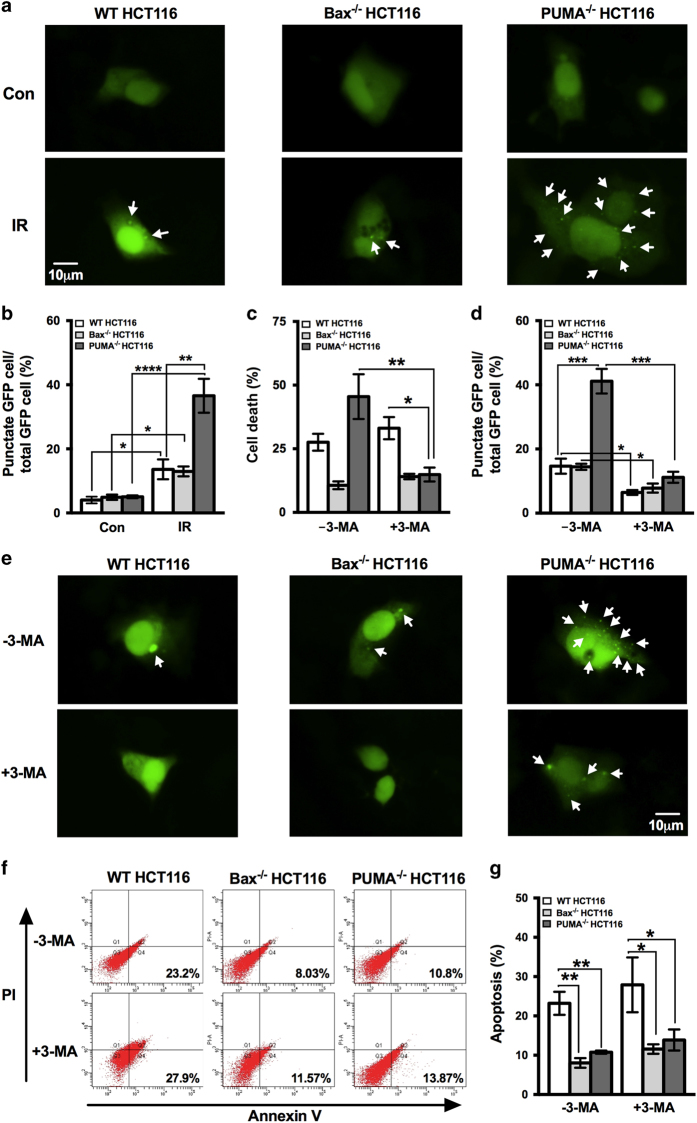
Examination of autophagy and inhibition of irradiation-induced autophagy in WT, Bax^−/−^ and PUMA^−/−^ HCT116 cells by 3-MA. WT, Bax^−/−^ and PUMA^−/−^ HCT116 cells were transfected with GFP-tagged LC3 plasmids. Twenty-four hours later, the transfected cells were subjected to irradiation of 8 Gy. The expression of GFP-LC3 was then examined by a fluorescence microscopy after 72 h incubation. Representative images are shown in (**a**) and the percentage of cells with punctate GFP-LC3 was calculated relative to all GFP-LC3 positive cells and shown in (**b**). GFP-LC3 puncta were pointed out by white arrows. WT, Bax^−/−^ and PUMA^−/−^ HCT116 cells that were transiently expressing GFP-LC3 were incubated in the presence or absence of 3-MA and then exposed to irradiation of 8 Gy. Seventy-two hours later, images were taken by a fluorescent microscope and the percentage of cells with punctate GFP were calculated (**d** and **e**). White arrow marks the GFP-LC3 puncta. WT, Bax^−/−^ and PUMA^−/−^ HCT116 cells were cultured with or without 3-MA and then subjected to irradiation of 8 Gy. Cell death (**c**) and apoptosis (**f** and **g**) were determined at 72 h after irradiation by CCK-8 kit and Annexin V-FITC staining with flow cytometry, respectively. Con, control, no irradiation. IR, irradiation. Statistical significant differences are marked as *P<0.05, **P<0.01, ***P<0.001 or ****P<0.0001.

Excluded the contribution of apoptosis, we tested the involvement of autophagy as autophagy is another important type of cell death. GFP-LC3 plasmids were used to examine the autophagy activity. All three cell lines, including WT, Bax^−/−^ and PUMA^−/−^ HCT116, showed significant increase in the percentage of cells with punctate GFP after irradiation (Figures 3a and b), indicating that autophagy was induced by irradiation in all three cell lines. This universal occurrence of autophagy after irradiation is quite consistent with the previous studies, which showed that autophagy was induced by radiation administration regardless of radiosensitivity or its pro-survival or pro-death role.^20,39^ On the other hand, the percentage of cells with punctate GFP in PUMA^−/−^ HCT116 was significantly higher than the other two cell lines WT and Bax^−/−^ HCT116 (Figures 3a and b). As excessive levels of autophagy are associated with autophagy-dependent cell death,^40^ the significantly more active autophagy in PUMA^−/−^ cells suggests that it is probably autophagy that leads to increased cell death.

In order to test this hypothesis, we blocked autophagy by its inhibitor 3-methyladenine (3-MA) and then determined cell death caused by irradiation again. When exposed to 3-MA, cell death of WT or Bax^−/−^ HCT116 cells did not change; however, suppressed autophagy significantly decreased the cell death caused by irradiation in PUMA^−/−^ cells (Figure 3c), which suggests that autophagy is likely the biological process that is responsible for the increased cell death in PUMA^−/−^ cells after irradiation. Before we jump to the conclusion, we also confirmed the efficiency of 3-MA by determining the autophagic activity after treatment of 3-MA. The percentage of cells with punctate GFP was significantly decreased by 3-MA (Figures 3d and e), indicating the successful inhibition of autophagy by 3-MA. Meanwhile, we also measured apoptosis after 3-MA exposure in order to make sure that apoptosis did not contribute to the decreased cell death. It was shown that apoptosis induced by irradiation before and after the treatment of 3-MA did not change (Figures 3f and g), which excludes the contribution of apoptosis in the decreased cell death as a result of autophagy inhibition. Based on all the data above, we conclude that autophagic cell death is induced by irradiation in PUMA^−/−^ HCT116 cells, which contributes to the increased cell death compared with WT cells. More importantly, this shows that PUMA is involved in the regulation of autophagic cell death.

**Figure 4:**
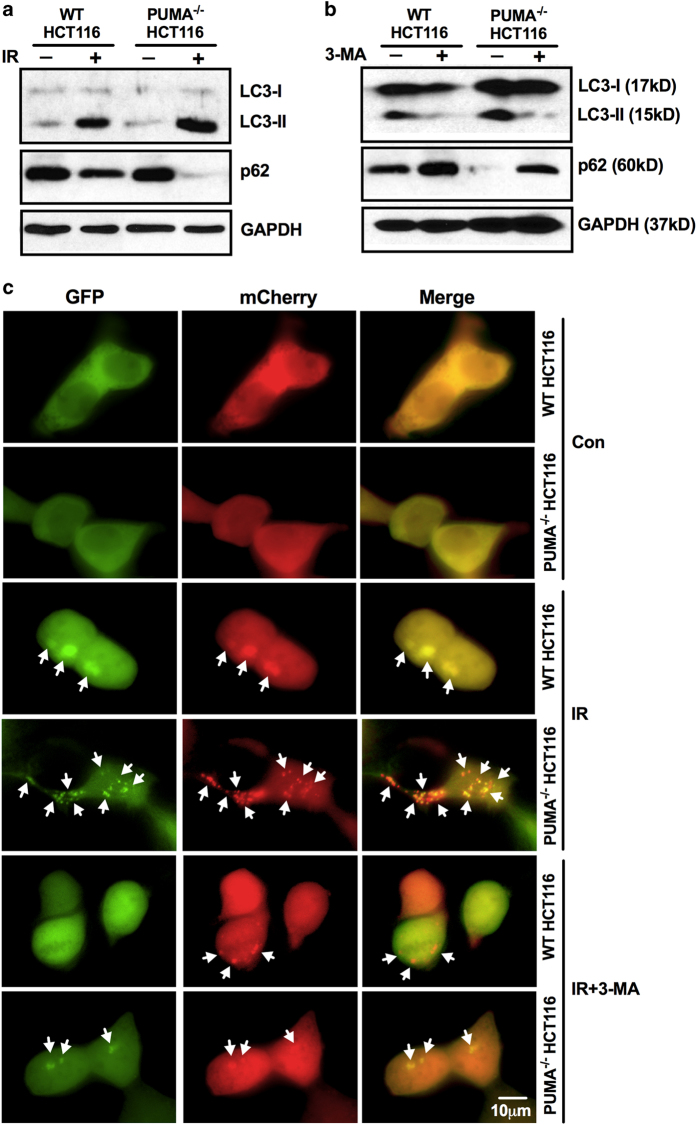
Autophagic flux in WT and PUMA^−/−^ HCT116 cells. (**a**) WT and PUMA^−/−^ HCT116 cells were subjected to irradiation of 8 Gy and proteins were extracted 72 h later. The protein level of LC3-I/II and p62 were determined by western blotting. Glyceraldehyde 3-phosphate dehydrogenase (GAPDH) was used as an internal control. (**b**) WT and PUMA^−/−^ HCT116 cells were exposed to irradiation of 8 Gy in the presence of 3-MA, and proteins were collected 72 h later for the determination of LC3-I/II and p62 protein level. GAPDH was used as internal control. (**c**) mCherry-GFP-LC3 plasmids were transfected into WT and PUMA^−/−^ HCT116 cells before the treatment by 3-MA, and then the cells were subjected to irradiation of 8 Gy. After 72 h incubation, images were taken by fluorescent microscope and mCherry and/or GFP puncta are pointed by white arrows.

To further confirm the involvement of PUMA in autophagy regulation, western blotting was performed to determine the protein level of LC3-I/II and p62, which are markers of autophagy. As shown in Figure 4a, irradiation significantly increased the expression of LC3-II that shows on autophagosome and decreased the accumulation of p62, a long-lived protein which inversely correlates with autophagic degradation, suggesting the irradiation-induced autophagy in WT and PUMA^−/−^ HCT116 cells. Moreover, compared with WT, PUMA^−/−^ HCT116 cells showed a significantly higher level of LC3-II but a significantly lower level of p62, which indicates the accelerated autophagy when PUMA is knocked out. This is confirmed by the decrease of LC3-II and increase of p62 protein level when autophagy was inhibited by 3-MA (Figure 4b). In order to confirm the autophagic flux in PUMA^−/−^ HCT116 cells upon irradiation with an independent method, mCherry-GFP-LC3 plasmids were introduced to demonstrate the fusion of autophagosome and lysosome. This plasmid loses GFP fluorescence in acidic condition when autophagosome fuses with lysosome to form acidified autolysosome, so the formation of autolysosome can be identified by the transition from showing both mCherry and GFP signals (yellow, autophagosome) to only showing mCherry (red, autolysosome).^41,42^ Punctate mCherry/GFP-LC3 increased upon radiation, and PUMA^−/−^ HCT116 cells showed significantly more puncta and the number decreased by 3-MA (Figure 4c). Both yellow and red dots showed up in WT and PUMA^−/−^ HCT116 cells, indicating the fusion of autophagosome and lysosome.

### Autophagic cell death also occurs in Bid^−/−^ MEF cells upon irradiation

**Figure 5:**
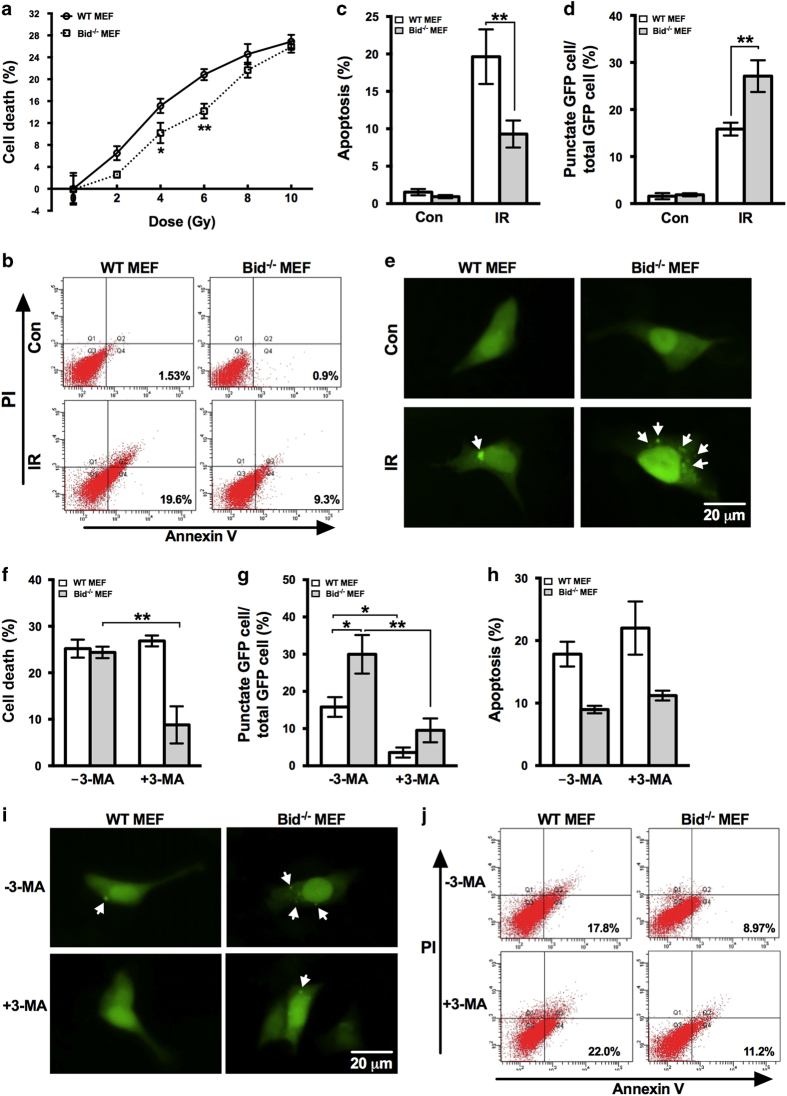
Autophagic cell death in Bid^−/−^ MEFs in response to irradiation. (**a**) WT and Bid^−/−^ MEFs were subjected to irradiation of the indicated doses. Seventy-two hours later, cell death was measured by CCK-8 kit and shown as means±S.D. (**b**) WT and Bid^−/−^ MEFs were exposed to 10 Gy of irradiation, and at 72 h after irradiation, apoptosis was assessed by Annexin-V and PI staining with flow cytometry. Representative flow cytometric plots are shown. (**c**) Quantification of apoptosis was shown as means±S.D. (**d** and **e**) WT and Bid^−/−^ MEFs were transfected with GFP-LC3 plasmids, and 24 h later, the transfected cells were subjected to irradiation of 10 Gy. After 72 h incubation, the expression of GFP-LC3 was examined by a fluorescent microscopy and the percentage of cells with punctate GFP was calculated. White arrow marks the GFP-LC3 puncta. (**f**) WT and Bid^−/−^ MEFs were incubated with or without 3-MA and then subjected to irradiation of 10 Gy, and cell death was determined 72 h later by CCK-8 kit. (**h** and **j**) Apoptosis was also measured by Annexin V-FITC staining with flow cytometry. (**g** and **i**) WT and Bid^−/−^ MEFs that were transiently expressing GFP-LC3 were cultured in the presence or absence of 3-MA and then exposed to irradiation of 10 Gy. Seventy-two hours after irradiation, images were taken and the percentage of cells with punctate GFP was calculated. White arrow marks the GFP-LC3 puncta. IR, irradiation; NT, non-treatment. Statistical significant differences are marked as *P<0.05 or **P<0.01.

It is known that the role of autophagy being pro-survival or pro-death is cell line and context dependent, so in order to test whether the autophagic cell death induced by irradiation is HCT116 cell specific or PUMA knockout specific, we introduced another BH3-only-protein-deficient cell line Bid^−/−^ MEF cells into the model. Both WT and Bid^−/−^ MEFs were subjected to irradiation of different doses and cell death was measured afterwards. Compared with WT MEFs, cell death in Bid^−/−^ cells was less at lower doses of irradiation, including 4 and 6 Gy (Figure 5a). However, the difference disappeared as irradiation dose increased and the cell death of WT and Bid^−/−^ MEFs at 10 Gy were relatively comparable (Figure 5a). As Bid is a proapoptotic gene, knockout of Bid would decrease cell death because apoptotic pathway is partially impaired, which is the case of Bid^−/−^ MEFs at lower doses, but this cannot explain the comparable cell death in WT and Bid^−/−^ MEFs at higher doses. Apoptosis was then measured in both cell lines to confirm the impairment of apoptotic pathway as a result of Bid deficiency. Irradiation of 10 Gy was used in experiments thereafter as Bid^−/−^ MEFs showed the least difference from WT cells at this dose. As expected, apoptosis induced by irradiation in Bid^−/−^ MEFs was significantly less than in WT cells (Figures 5b and c). Confirmed the significantly decreased apoptosis in Bid^−/−^ MEFs, we hypothesized that autophagic cell death may contribute to cell death induced by irradiation in Bid^−/−^ MEFs.

In order to test this hypothesis, we first detected the autophagy activity of Bid^−/−^ MEFs after irradiation by the expression of GFP-LC3. The percentage of cells with punctate GFP in Bid^−/−^ MEFs was significantly higher than that of WT cells although both of them showed increase (Figures 5d and e), which, to some extent, supports the hypothesis. Then, in order to demonstrate the involvement of autophagic cell death, 3-MA was used to block autophagy and Bid^−/−^ MEFs showed significant decrease in the percentage of cells expressing punctate GFP, as well as WT cells (Figures 5g and i). By measuring cell death after autophagy inhibition, Bid^−/−^ MEFs showed significant decrease as a result of 3-MA treatment while WT MEFs did not show any change (Figure 5f), suggesting the occurrence of autophagic cell death induced by irradiation in Bid^−/−^ MEFs, which contributes to the total cell death of Bid^−/−^ MEFs when apoptosis was impaired and makes the cell death between Bid^−/−^ and WT MEFs comparable. Apoptosis of WT and Bid^−/−^ MEFs with or without 3-MA were also determined, and neither of them showed any significant change (Figures 5h and j), which further confirmed the contribution of autophagic cell death in Bid^−/−^ MEFs. The results above suggest that, similar to PUMA, Bid is also involved in the regulation of autophagic cell death at least upon irradiation.

## Discussion

Autophagy is an important intracellular pathway for degradation and recycling of proteins and organelles, which can be induced by radiation either to protect cells against radiation damage or lead to cell death.^20^ However, the machinery system that controls whether autophagy is an adaptive mechanism to prevent cell death or a way to precede death is unclear. This study identified that autophagic cell death was activated in cells deficient of PUMA or Bid, which suggests the involvement of Bcl-2 family proteins PUMA and Bid in regulating autophagy toward cell death.

Autophagy has been observed in causing cell death in response to toxins, chemotherapeutic drugs and irradiation.^20,43–45^ In our study, autophagic cell death was found in PUMA^−/−^ HCT116 cells and Bid^−/−^ MEFs after irradiation (Figure 1 and Figure 4). However, we would like to mention that autophagic cell death was only shown at higher doses of irradiation but not at lower doses, indicating the dose dependence. In addition, the considerably significant increase in cell death of PUMA^−/−^ HCT116 cells and Bid^−/−^ MEFs, compared with WT cells, was not shown until 48 h postirradiation (Figure 1, Figure 4 and data not shown), which indicates the time dependence. This dose and time dependence suggest that autophagic cell death may be a response to only severe stresses and needs time to accumulate the degradation of substrates until elimination of essential components. On the other hand, these data do not argue against the protective role of autophagy. We cannot tell whether there is co-occurence of both pro-survival and pro-death autophagy at lower doses of irradiation. The decision about autophagy to promote survival or death might be made after its the initiation because acidic vesicular organelles (AVO) were present in both radiosensitive and redioresistant glioblastoma cell lines, which indicates that autophagic cell death happens after the formation of AVO.^39^ As well as PUMA^−/−^ HCT116 and Bid^−/−^ MEFs, autophagy was also significantly induced in WT and Bax^−/−^ HCT 116 and WT MEFs, but inhibition of autophagy in those cells did not change the cell death (Figures 3a–e and 4d–i), so the role of autophagy in those cells upon irradiation is unclear, which needs further pursuing to identify.

On contrary to our results, Tsujimoto and Shimizu^46^ found that X-ray irradiation was not able to induce autophagic cell death in Bax/Bak-deficient MEFs. As irradiation-inducing autophagic cell death was reported by multiple groups including us,^20,39,47–49^ it is possible that activation of autophagic cell death depends not only on death stinuli but also on cell line, genetic background and the context. Instead of one single decisive factor, it is rather a complex of multiple components. This also explains that Bcl-2 overexpression did not sensitize HCT116, Hela and Jurkat cells to autophagic cell death induced by etoposide although it did in Bax/Bak^−/−^ MEFs and Bax/Bak^−/−^ thymocytes,^22^ but HCT116 cells did show autophagic cell death induced by irradiation when they were PUMA^−/−^. Cargo specificity was also shown to be critical in controling the pro-survival or pro-death role of autophagy.^50^

In order to clarify the mechanism behind autophagic cell death, the causative relationship between apoptosis inhibition and autophagic cell death has been debated. Studies showed that autophagic cell death occurs when apoptosis is inhibited, either by pan-caspase inhibitor zVAD or as a result of proapoptotic genes Bax and Bak deficiency. Blocking apoptosis with zVAD or Bax/Bak siRNA radiosensitized cancer cells and increased cytotoxicity via autophagy in breast, lung and prostate cancer.^47,49^ When exposed to z-VAD, L929 cells died with ROS accumulation and loss of plasma membrane integrity, which was inhibited by autophagic inhibitors or knocking down of key autophagic genes (Atg7 or Atg8), indicating autophagic cell death in non-apoptotic cells.^50^ Bax/Bak^−/−^ MEFs were shown more radiosensitive than WT cells, with significant increase of autophagic proteins Atg5-Atg12 complex and Beclin-1, and suppressing autophagy by 3-MA or siAtg5/Beclin1 oppositely rendered radioresistance, which strongly suggests the autophagic cell death in Bax/Bak^−/−^ MEFs.^48^ These studies seem to support the simply causative relationship between apoptosis inhibition and autophagic cell death; however, overexpressing Atg5 and Beclin1 also conveyed radiosensitivity to WT MEFs by inducing autophagy although apoptosis program in WT MEFs is normal.^48^ Furthermore, although exposed to zVAD, autophagic cell death was not activated in WT, Apaf-1- or caspase-9-deficient MEFs.^22^ Therefore, autophagic cell death is not simply caused by impaired apoptosis program, which supports our result that autophagic cell death was not detected in Bax^−/−^ HCT116 cells. This is also consistent with the result that Bak-transfected Bax/Bak^−/−^ MEFs showed features of apoptosis, which was suppressed by zVAD but not 3-MA.^22^ Inhibition of autophagy alone is not sufficient to induce autophagic cell death; conversely, it may activate autophagy to promote survival. Autophagy was induced in bone marrow-derived cells from Bax/Bck^−/−^ mice, and autophagy was shown to be essential for their survival after growth factor withdrawal for at least 6 weeks.^26^

Our results suggest the involvement of PUMA and Bid in autophagic cell death regulation, but how are they involved is not clear yet. Bcl-2 is a critical determinant that drives cells toward apoptosis or autophagy.^51^ Bcl-2 or Bcl-xl overexpression induced autophagic cell death in WT MEFs and autophagic cell death in Bax/Bak^−/−^ MEFs was also modulated by Bcl-2 and Bcl-xL,^22^ so it is possible that PUMA and Bid is involved in autophagic cell death regulation through their interaction with Bcl-2 or Bcl-xL. Antiapoptotic Bcl-2 family proteins such as Bcl-2 and Bcl-xL inhibit apoptosis by forming heterodimers with proapoptotic members including PUMA and Bid to neutralize their action,^52^ so deficiency of PUMA or Bid relatively releases Bcl-2 or Bcl-xL, which can drive autophagy toward cell death. Bcl-2 or Bcl-xl may modulate autophagic cell death induced by irradiation via Beclin 1 as Beclin 1 was shown required for autophagic cell death and Bcl-2/Bcl-xl binds to Beclin 1 but not Bax/Bak.^53^ Another alternative explanation for the involvement of PUAM and Bid in regulating autophagic cell death might be their BH3-only protein characteristics. BH3-only proteins have a key role in promoting apoptosis: apoptotic stimuli induce expression and/or activation of BH3-only proteins, which translocate to mitochondria and activate Bax/Bak-dependent apoptosis.^54^ Preclinical data suggest that BH3-only proteins are important mediators in response to DNA damage, which is the main consequence of irradiation.^55^ More importantly, (−)-gossypol, a natural BH3-mimetic (also a Bcl-2 inhibitor) induced comparable levels of cell death in prostate cancer cell lines regardless of their Bcl-2 expression.^56^ However, in cell lines with low Bcl-2, >80% cells die through apoptosis, which was blocked by zVAD, while >60% cells with high Bcl-2 died by autophagy, which was blocked by 3-MA or Atg5/Beclin1 siRNA,^56^ suggesting the importance of BH3 motif and their interaction with Bcl-2 in regulation of autophagic cell death. BH3-only proteins were also mentioned in study by Tsujimoto group, which said that overexpression of BH3 proteins did not induce autophagic cell death in Bax/Bak^−/−^ MEFs.^22^ The rationale probably is, instead of overexpressing them, deleting or suppressing BH3 proteins should have been tried. One more alternative pathway through which PUMA and Bid are involved in autophagic cell death regulation might be p53 signaling as PUMA and Bid are both direct target genes of p53.^57–59^ However, there is rarely any supportive evidence. Pro-surviving autophagy rather than autophagic cell death was shown in tumor cells that lack p53,^60^ and some report said mutant p53 had no effect on accumulation of autophagosomes.^28^

Our previous study showed hypoxia-induced autophagy promoting survival and chemo-resistance in liver cancer cell lines,^61^ but this study showed the dose- and time- dependent autophagic cell death induced by irradiation, which suggests the debatable treatment by targeting autophagy in cancer. More systematic and comprehensive investigations are needed to evaluate the application of antiautophagy or pro-autophagy treatment in translational studies. Clinical trial selection criteria studies should be designed more carefully with genomic information in mind as the genotype may totally change the role of autophagy. Combination of the autophagic activity and the genomic background of patient are necessary to assess the applicability of antiautophagy or pro-autophagy treatment in cancer. It is critical to find the check point(s) that control the change of the role of autophagy from pro-survival to pro-death. Most tumors have certain defects in apoptosis signaling pathway, so cautiously manipulating autophagy may yield promising clinical outcomes for patients undergoing RT and chemotherapy. Some tumors such as gliomas are resistant to apoptotic stimuli but sensitive to autophagic cell death, which indicates that the effectiveness of RT in specific subtypes of tumors may strongly depend on autophagy.^62^ Elucidating the check point(s) and their effect(s) in mediating radio-resistance or -sensitivity may allow physicians to make personalized therapy according to patients’ genomic background. Therefore, identifying the molecular mechanism by which deficiency of certain proapoptosis gene(s) increases radiosensitivity will help develop novel efficient strategies for cancer patients of specific genotype.

## Materials and methods

### Cell culture and treatments

Human colon cancer cell lines: WT, Bid^−/−^ and PUMA^−/−^ HCT116, and MEFs: WT and Bid^−/−^ MEFs, were maintained in Dulbecco’s modified Eagle’s medium of high glucose (DMEM, GIBCO, Invitrogen, Shanghai, China) and supplemented with 10% fetal bovine serum (GIBCO, Invitrogen), 100 units/ml penicillin and 100 mg/ml streptomycin in a humidified incubator under 95% air and 5% CO_2_ at 37 °C. X-ray irradiation was performed by Elekta Precise accelerator (Crawley, England). Dimethyl sulfoxide and 3-MA were purchased from Sigma-Aldrich (Shanghai, China), and 5 mM 3-MA was used in experiments.

### Cell death assay

Cell viability was determined with Cell Counting Kit-8 (CCK-8, Dojindo, Tokyo, Japan). Briefly, at the end of the treatment, CCK-8 solution was added to 96-well plate with cultured cells followed by 2 h incubation at 37 °C. Then the absorbance of each well was measured with a microplate reader (Synergy HT, Bio-Tek, Winooski, VT, USA) at 450 nm and the percentage of dead cells was calculated based on the absorbance.

### Apoptosis assay

Apoptosis examination by Annexin V-FITC assay with flow cytometry was performed based on the manufacturer’s instructions (Nanjing Keygen Biotech, Nanjing, China). Briefly, cells were collected by trypsinization, washed with ice-cold phosphate-buffered saline twice and resuspended in 300 *μ*l 1× binding buffer containing 5 *μ*l Annexin V and 5 *μ*l propidium iodide (PI) followed by incubation in the dark for 30 min at room temperature. Then ⩾10 000 cells were analyzed on a BD FACSAria flow cytometer (Becton Dickinson, Franklin Lakes, NJ, USA). Annexin V-positive and PI-negative cells were considered as apoptotic cells.

### Transient transfection and identification of autophagy

GFP-tagged microtubule-associated protein 1 light chain 3 (LC3) expression plasmids and mCherry-GFP-LC3 plasmids were transfected to determine autophagy. Briefly, plasmids were transiently transfected into cells by Fugene HD transfection reagent (Roche, Madison, WI, USA) according to the manufacturer’s instructions. The transfected cells were then incubated for 24 h to ensure the expression of targeted protein in the plasmids. Afterwards, cells were subjected to the indicated treatments. At the end of the treatment, cells were observed under fluorescent microscope (Olympus IX71, Olympus, Center Valley, PA, USA) and cells with punctate GFP or mCherry were identified as autophagic cells. In GFP-LC3-transfected cells, in order to quantify autophagic activity, cells with GFP and cells with punctate GFP were counted, respectively, and autophagy activity was calculated by the percentage of cells with GFP-LC3-positive dots. A minimum of 200 cells were counted for each sample.

### Western blotting

Protein was extracted from cells by RIPA lysis buffer (Beyotime, Haimen, China, Cat. no. P0013B) with 1 mM PMSF. Equal amounts of protein was separated by SDS-PAGE and transferred onto NC membrane. After blocking with 5% non-fat milk, the membrane was probed with anti-p62 (Novus Biologicals Inc., Littleton, CO, USA Cat. no. NBP1-48320) and anti-LC3 (Novus Biologicals Inc. Cat. no. NB100-2220H), developed with the BeyoECL Plus substrate system (Beyotime, Cat. no. P0018). Anti-GAPDH (Cell Signaling, Danvers, MA, USA, Cat. no. 2118) was used as an internal control to confirm equal protein loading.

### Statistical analysis

All experiments were repeated for at least three times. Data were expressed as mean±S.D. Statistical analysis was performed by two-way ANOVA and unpaired Student’s *T*-test. *P*<0.05 was considered significant.

## Additional information

**Publisher’s note:** Springer Nature remains neutral with regard to jurisdictional claims in published maps and institutional affiliations.

## References

[bib1] Connell PP, Hellman S. Advances in radiotherapy and implications for the next century: a historical perspective. Cancer Res 2009; 69: 383–392. 19147546 10.1158/0008-5472.CAN-07-6871

[bib2] Fokas E, Rodel C. Targeted agents in GI radiotherapy: clinical efficacy and side effects. Best Pract Res Clin Gastroenterol 2016; 30: 537–549. 27644903 10.1016/j.bpg.2016.05.002

[bib3] Schaue D, McBride WH. Opportunities and challenges of radiotherapy for treating cancer. Nat Rev Clin Oncol 2015; 12: 527–540. 26122185 10.1038/nrclinonc.2015.120 PMC8396062

[bib4] Delaney G, Jacob S, Featherstone C, Barton M. The role of radiotherapy in cancer treatment: estimating optimal utilization from a review of evidence-based clinical guidelines. Cancer 2005; 104: 1129–1137. 16080176 10.1002/cncr.21324

[bib5] Chadwick KH, Leenhouts HP. The Molecular Theory of Radiation biology. Monographs of Theoretical and Applied Genetics 5. Springer Verlag: Berlin, Heidelberg, Germany, 1981, pp 143–181.

[bib6] Verma V, Moreno AC, Lin SH. Advances in radiotherapy management of esophageal cancer. J Clin Med 2016; 5: 91. 10.3390/jcm5100091 PMC5086593 27775643

[bib7] Hanks GE, Pajak TF, Porter A, Grignon D, Brereton H, Venkatesan V et al. Shipley WU and Radiation Therapy Oncology Group. Phase III trial of long-term adjuvant androgen deprivation after neoadjuvant hormonal cytoreduction and radiotherapy in locally advanced carcinoma of the prostate: the Radiation Therapy Oncology Group Protocol 92-02. J Clin Oncol 2003; 21: 3972–3978. 14581419 10.1200/JCO.2003.11.023

[bib8] Dumont FJ, Bischoff P. Disrupting the mTOR signaling network as a potential strategy for the enhancement of cancer radiotherapy. Curr Cancer Drug Targets 2012; 12: 899–924. 22831276 10.2174/156800912803251243

[bib9] Kuban DA, el-Mahdi AM, Schellhammer PF. Effect of local tumor control on distant metastasis and survival in prostatic adenocarcinoma. Urology 1987; 30: 420–426. 3118547 10.1016/0090-4295(87)90372-4

[bib10] Lowe SW, Ruley HE, Jacks T, Housman DE. p53-dependent apoptosis modulates the cytotoxicity of anticancer agents. Cell 1993; 74: 957–967. 8402885 10.1016/0092-8674(93)90719-7

[bib11] Tsujimoto Y. Cell death regulation by the Bcl-2 protein family in the mitochondria. J Cell Physiol 2003; 195: 158–167. 12652643 10.1002/jcp.10254

[bib12] Strasser A, Cory S, Adams JM. Deciphering the rules of programmed cell death to improve therapy of cancer and other diseases. EMBO J 2011; 30: 3667–3683. 21863020 10.1038/emboj.2011.307 PMC3173800

[bib13] Muchmore SW, Sattler M, Liang H, Meadows RP, Harlan JE, Yoon HS et al. X-ray and NMR structure of human Bcl-xL, an inhibitor of programmed cell death. Nature 1996; 381: 335–341. 8692274 10.1038/381335a0

[bib14] Adams JM, Cory S. The Bcl-2 protein family: arbiters of cell survival. Science 1998; 281: 1322–1326. 9735050 10.1126/science.281.5381.1322

[bib15] Wang K, Yin XM, Chao DT, Milliman CL, Korsmeyer SJ. BID: a novel BH3 domain-only death agonist. Genes Dev 1996; 10: 2859–2869. 8918887 10.1101/gad.10.22.2859

[bib16] Garcia-Saez AJ. The secrets of the Bcl-2 family. Cell Death Differ 2012; 19: 1733–1740. 22935609 10.1038/cdd.2012.105 PMC3469065

[bib17] Rampino N, Yamamoto H, Ionov Y, Li Y, Sawai H, Reed JC et al. Somatic frameshift mutations in the BAX gene in colon cancers of the microsatellite mutator phenotype. Science 1997; 275: 967–969. 9020077 10.1126/science.275.5302.967

[bib18] Meijerink JP, Mensink EJ, Wang K, Sedlak TW, Sloetjes AW, de Witte T et al. Hematopoietic malignancies demonstrate loss-of-function mutations of BAX. Blood 1998; 91: 2991–2997. 9531611

[bib19] Verheij M, Bartelink H. Radiation-induced apoptosis. Cell Tissue Res 2000; 301: 133–142. 10928286 10.1007/s004410000188

[bib20] Paglin S, Hollister T, Delohery T, Hackett N, McMahill M, Sphicas E et al. A novel response of cancer cells to radiation involves autophagy and formation of acidic vesicles. Cancer Res 2001; 61: 439–444. 11212227

[bib21] Yu L, Alva A, Su H, Dutt P, Freundt E, Welsh S et al. Regulation of an ATG7-beclin 1 program of autophagic cell death by caspase-8. Science 2004; 304: 1500–1502. 15131264 10.1126/science.1096645

[bib22] Shimizu S, Kanaseki T, Mizushima N, Mizuta T, Arakawa-Kobayashi S, Thompson CB et al. Role of Bcl-2 family proteins in a non-apoptotic programmed cell death dependent on autophagy genes. Nat Cell Biol 2004; 6: 1221–1228. 15558033 10.1038/ncb1192

[bib23] Bursch W. The autophagosomal-lysosomal compartment in programmed cell death. Cell Death Differ 2001; 8: 569–581. 11536007 10.1038/sj.cdd.4400852

[bib24] Gozuacik D, Kimchi A. Autophagy as a cell death and tumor suppressor mechanism. Oncogene 2004; 23: 2891–2906. 15077152 10.1038/sj.onc.1207521

[bib25] Hippert MM, O'Toole PS, Thorburn A. Autophagy in cancer: good, bad, or both? Cancer Res 2006; 66: 9349–9351. 17018585 10.1158/0008-5472.CAN-06-1597

[bib26] Lum JJ, Bauer DE, Kong M, Harris MH, Li C, Lindsten T et al. Growth factor regulation of autophagy and cell survival in the absence of apoptosis. Cell 2005; 120: 237–248. 15680329 10.1016/j.cell.2004.11.046

[bib27] Boya P, Gonzalez-Polo RA, Casares N, Perfettini JL, Dessen P, Larochette N et al. Inhibition of macroautophagy triggers apoptosis. Mol Cell Biol 2005; 25: 1025–1040. 15657430 10.1128/MCB.25.3.1025-1040.2005 PMC543994

[bib28] Apel A, Herr I, Schwarz H, Rodemann HP, Mayer A. Blocked autophagy sensitizes resistant carcinoma cells to radiation therapy. Cancer Res 2008; 68: 1485–1494. 18316613 10.1158/0008-5472.CAN-07-0562

[bib29] Chaachouay H, Ohneseit P, Toulany M, Kehlbach R, Multhoff G, Rodemann HP. Autophagy contributes to resistance of tumor cells to ionizing radiation. Radiother Oncol 2011; 99: 287–292. 21722986 10.1016/j.radonc.2011.06.002

[bib30] Bergmann A. Autophagy and cell death: no longer at odds. Cell 2007; 131: 1032–1034. 18083090 10.1016/j.cell.2007.11.027 PMC2502067

[bib31] Alva AS, Gultekin SH, Baehrecke EH. Autophagy in human tumors: cell survival or death? Cell Death Differ 2004; 11: 1046–1048. 15143348 10.1038/sj.cdd.4401445

[bib32] Bae H, Guan JL. Suppression of autophagy by FIP200 deletion impairs DNA damage repair and increases cell death upon treatments with anticancer agents. Mol Cancer Res 2011; 9: 1232–1241. 21807966 10.1158/1541-7786.MCR-11-0098 PMC3175275

[bib33] Leist M, Jaattela M. Four deaths and a funeral: from caspases to alternative mechanisms. Nat Rev Mol Cell Biol 2001; 2: 589–598. 11483992 10.1038/35085008

[bib34] Kroemer G, Marino G, Levine B. Autophagy and the integrated stress response. Mol Cell 2010; 40: 280–293. 20965422 10.1016/j.molcel.2010.09.023 PMC3127250

[bib35] Qu X, Yu J, Bhagat G, Furuya N, Hibshoosh H, Troxel A et al. Promotion of tumorigenesis by heterozygous disruption of the beclin 1 autophagy gene. J Clin Invest 2003; 112: 1809–1820. 14638851 10.1172/JCI20039 PMC297002

[bib36] Yue Z, Jin S, Yang C, Levine AJ, Heintz N. Beclin 1, an autophagy gene essential for early embryonic development, is a haploinsufficient tumor suppressor. Proc Natl Acad Sci USA 2003; 100: 15077–15082. 14657337 10.1073/pnas.2436255100 PMC299911

[bib37] Kondo Y, Kanzawa T, Sawaya R, Kondo S. The role of autophagy in cancer development and response to therapy. Nat Rev Cancer 2005; 5: 726–734. 16148885 10.1038/nrc1692

[bib38] Maiuri MC, Zalckvar E, Kimchi A, Kroemer G. Self-eating and self-killing: crosstalk between autophagy and apoptosis. Nat Rev 2007; 8: 741–752. 10.1038/nrm2239 17717517

[bib39] Yao KC, Komata T, Kondo Y, Kanzawa T, Kondo S, Germano IM. Molecular response of human glioblastoma multiforme cells to ionizing radiation: cell cycle arrest, modulation of the expression of cyclin-dependent kinase inhibitors, and autophagy. J Neurosurg 2003; 98: 378–384. 12593626 10.3171/jns.2003.98.2.0378

[bib40] Liu Y, Levine B. Autosis and autophagic cell death: the dark side of autophagy. Cell Death Differ 2015; 22: 367–376. 25257169 10.1038/cdd.2014.143 PMC4326571

[bib41] Kimura S, Noda T, Yoshimori T. Dissection of the autophagosome maturation process by a novel reporter protein, tandem fluorescent-tagged LC3. Autophagy 2007; 3: 452–460. 17534139 10.4161/auto.4451

[bib42] Elrick MJ, Lieberman AP. Autophagic dysfunction in a lysosomal storage disorder due to impaired proteolysis. Autophagy 2013; 9: 234–235. 23086309 10.4161/auto.22501 PMC3552886

[bib43] Talloczy Z, Jiang W, HWt Virgin, Leib DA, Scheuner D, Kaufman RJ et al. Regulation of starvation- and virus-induced autophagy by the eIF2alpha kinase signaling pathway. Proc Natl Acad Sci USA 2002; 99: 190–195. 11756670 10.1073/pnas.012485299 PMC117537

[bib44] Kanzawa T, Germano IM, Komata T, Ito H, Kondo Y, Kondo S. Role of autophagy in temozolomide-induced cytotoxicity for malignant glioma cells. Cell Death Differ 2004; 11: 448–457. 14713959 10.1038/sj.cdd.4401359

[bib45] Daido S, Kanzawa T, Yamamoto A, Takeuchi H, Kondo Y, Kondo S. Pivotal role of the cell death factor BNIP3 in ceramide-induced autophagic cell death in malignant glioma cells. Cancer Res 2004; 64: 4286–4293. 15205343 10.1158/0008-5472.CAN-03-3084

[bib46] Tsujimoto Y, Shimizu S. Another way to die: autophagic programmed cell death. Cell Death Differ 2005; 12(Suppl 2): 1528–1534. 16247500 10.1038/sj.cdd.4401777

[bib47] Cao C, Subhawong T, Albert JM, Kim KW, Geng L, Sekhar KR et al. Inhibition of mammalian target of rapamycin or apoptotic pathway induces autophagy and radiosensitizes PTEN null prostate cancer cells. Cancer Res 2006; 66: 10040–10047. 17047067 10.1158/0008-5472.CAN-06-0802

[bib48] Kim KW, Mutter RW, Cao C, Albert JM, Freeman M, Hallahan DE et al. Autophagy for cancer therapy through inhibition of pro-apoptotic proteins and mammalian target of rapamycin signaling. J Biol Chem 2006; 281: 36883–36890. 17005556 10.1074/jbc.M607094200

[bib49] Moretti L, Kim KW, Jung DK, Willey CD, Lu B. Radiosensitization of solid tumors by Z-VAD, a pan-caspase inhibitor. Mol Cancer Ther 2009; 8: 1270–1279. 19417149 10.1158/1535-7163.MCT-08-0893 PMC2888880

[bib50] Yu L, Wan F, Dutta S, Welsh S, Liu Z, Freundt E et al. Autophagic programmed cell death by selective catalase degradation. Proc Natl Acad Sci USA 2006; 103: 4952–4957. 16547133 10.1073/pnas.0511288103 PMC1458776

[bib51] Lian J, Karnak D, Xu L. The Bcl-2-Beclin 1 interaction in (-)-gossypol-induced autophagy versus apoptosis in prostate cancer cells. Autophagy 2010; 6: 1201–1203. 20930561 10.4161/auto.6.8.13549 PMC3039723

[bib52] Llambi F, Moldoveanu T, Tait SW, Bouchier-Hayes L, Temirov J, McCormick LL et al. A unified model of mammalian BCL-2 protein family interactions at the mitochondria. Mol Cell 2011; 44: 517–531. 22036586 10.1016/j.molcel.2011.10.001 PMC3221787

[bib53] Liang XH, Kleeman LK, Jiang HH, Gordon G, Goldman JE, Berry G et al. Protection against fatal Sindbis virus encephalitis by beclin, a novel Bcl-2-interacting protein. J Virol 1998; 72: 8586–8596. 9765397 10.1128/jvi.72.11.8586-8596.1998 PMC110269

[bib54] Michael Kastan, Abeloff MDA, James O, Niederhuber John E. Abeloff's Clinical Oncology4th ednChurchill Livingstone/Elsevier: Philadelphia, PA, USA, 2008.

[bib55] Certo M, Del Gaizo Moore V, Nishino M, Wei G, Korsmeyer S, Armstrong SA et al. Mitochondria primed by death signals determine cellular addiction to antiapoptotic BCL-2 family members. Cancer Cell 2006; 9: 351–365. 16697956 10.1016/j.ccr.2006.03.027

[bib56] Lian J, Wu X, He F, Karnak D, Tang W, Meng Y et al. A natural BH3 mimetic induces autophagy in apoptosis-resistant prostate cancer via modulating Bcl-2-Beclin1 interaction at endoplasmic reticulum. Cell Death Differ 2011; 18: 60–71. 20577262 10.1038/cdd.2010.74 PMC2950895

[bib57] Yu J, Wang Z, Kinzler KW, Vogelstein B, Zhang L. PUMA mediates the apoptotic response to p53 in colorectal cancer cells. Proc Natl Acad Sci USA 2003; 100: 1931–1936. 12574499 10.1073/pnas.2627984100 PMC149936

[bib58] Sax JK, Fei P, Murphy ME, Bernhard E, Korsmeyer SJ, El-Deiry WS. BID regulation by p53 contributes to chemosensitivity. Nat Cell Biol 2002; 4: 842–849. 12402042 10.1038/ncb866

[bib59] Miyashita T, Reed JC. Tumor suppressor p53 is a direct transcriptional activator of the human bax gene. Cell 1995; 80: 293–299. 7834749 10.1016/0092-8674(95)90412-3

[bib60] Amaravadi RK, Yu D, Lum JJ, Bui T, Christophorou MA, Evan GI et al. Autophagy inhibition enhances therapy-induced apoptosis in a Myc-induced model of lymphoma. J Clin Invest 2007; 117: 326–336. 17235397 10.1172/JCI28833 PMC1765515

[bib61] Song J, Qu Z, Guo X, Zhao Q, Zhao X, Gao L et al. Hypoxia-induced autophagy contributes to the chemoresistance of hepatocellular carcinoma cells. Autophagy 2009; 5: 1131–1144. 19786832 10.4161/auto.5.8.9996

[bib62] Zois CE, Koukourakis MI. Radiation-induced autophagy in normal and cancer cells: towards novel cytoprotection and radio-sensitization policies? Autophagy 2009; 5: 442–450. 19164950 10.4161/auto.5.4.7667

